# Haplotype association analysis of genes within the WNT signalling pathways in diabetic nephropathy

**DOI:** 10.1186/1471-2369-14-126

**Published:** 2013-06-18

**Authors:** David H Kavanagh, David A Savage, Christopher C Patterson, Amy Jayne McKnight, John K Crean, Alexander P Maxwell, Gareth J McKay

**Affiliations:** 1 Nephrology Research Group, Centre for Public Health, Queen’s University Belfast, Belfast BT12 6BJ, UK; 2 Histocompatibility & Immunogenetics Laboratory, Belfast Health and Social Care Trust, Belfast City Hospital, Belfast, UK; 3 Conway Institute, University College Dublin, Dublin, Ireland

**Keywords:** Diabetic nephropathy, WNT signalling pathway, Association study, End-stage renal disease

## Abstract

**Background:**

Renal interstitial fibrosis and glomerular sclerosis are hallmarks of diabetic nephropathy (DN) and several studies have implicated members of the WNT pathways in these pathological processes. This study comprehensively examined common genetic variation within the WNT pathway for association with DN.

**Methods:**

Genes within the WNT pathways were selected on the basis of nominal significance and consistent direction of effect in the GENIE meta-analysis dataset. Common SNPs and common haplotypes were examined within the selected WNT pathway genes in a white population with type 1 diabetes, discordant for DN (cases: n = 718; controls: n = 749). SNPs were genotyped using Sequenom or Taqman assays. Association analyses were performed using PLINK, to compare allele and haplotype frequencies in cases and controls. Correction for multiple testing was performed by either permutation testing or using false discovery rate.

**Results:**

A logistic regression model including collection centre, duration of diabetes, and average HbA1c as covariates highlighted three SNPs in *GSK3B* (rs17810235, rs17471, rs334543), two in *DAAM1* (rs1253192, rs1252906) and one in *NFAT5* (rs17297207) as being significantly (*P* < 0.05) associated with DN, however these SNPs did not remain significant after correction for multiple testing. Logistic regression of haplotypes, with ESRD as the outcome, and pairwise interaction analyses did not yield any significant results after correction for multiple testing.

**Conclusions:**

These results indicate that both common SNPs and common haplotypes of WNT pathway genes are not strongly associated with DN. However, this does not completely exclude these or the WNT pathways from association with DN, as unidentified rare genetic or copy number variants could still contribute towards the genetic architecture of DN.

## Background

Diabetic nephropathy (DN) is a microvascular complication of diabetes and is the most frequent cause of end-stage renal disease (ESRD) in western populations [1]. Renal interstitial fibrosis and glomerular sclerosis are histological hallmarks of DN and several studies have implicated members of the WNT pathways in these pathological processes [2-9].

**Figure 1:**
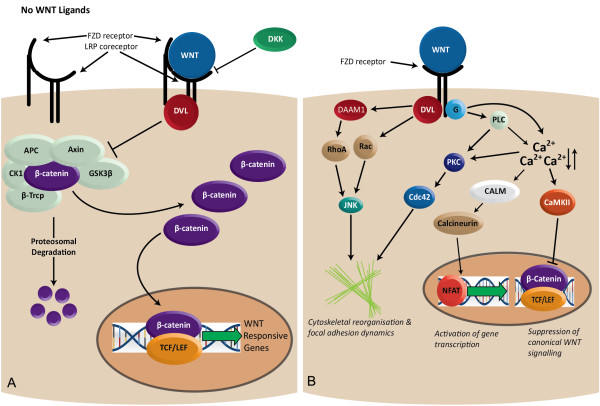
**Wnt signalling pathways implicated in diabetic nephropathy.** Eleven genes encoding members of the Wnt signalling pathway were prioritized for assessment of association with diabetic nephropathy (*AXIN1, CALM3, CTNNB1, DAAM1, DKK2, GSK3B, NFAT5, WNT3, WNT5A, WNT6* and *WNT16*). (**A**) Canonical WNT signalling: Some WNT ligands bind to FZD and LRP receptors. DKK internalises the LRP receptors blocking canonical WNT signalling. DVL recruits the AXIN GSK3B destruction complex that is responsible for marking B-catenin for proteosomal degradation. (**B**) Non-canonical WNT signalling: Certain WNT ligands bind to FZD receptors and elicit the activation of DVL and heterotrimeric G-proteins which in turn can activate DAAM which regulates cytoskeletal organization and focal adhesion dynamics. In addition, activation of CALM regulates the transcription factor NFAT and modulates gene expression.

The WNT pathways can be subdivided into canonical β-catenin dependent and non-canonical β-catenin independent pathways (Figure 1). Canonical WNT signalling is central to numerous developmental processes and variants discovered within members of this pathway have been implicated in multiple complex diseases such as familial adenomatous polyposis coli, colorectal and hepatocellular cancers, type 2 diabetes and schizophrenia [10-14]. Non-canonical WNT signalling is less well defined, in part due to further subdivisions into the WNT/Ca^2+^ and the WNT planar cell polarity pathways. These pathways have been shown to modulate cytoskeletal reorganisation and activation of the JNK and MAPK signalling pathways [15-17], both of which can affect the motility and adherence of the mesangial cell, perturbing the cell's response to dynamic mechanical forces, which is the key function of the mesangial cell.

*In vitro* epithelial-to-mesenchymal transition (EMT) promotes renal fibrosis and can be induced by TGF-β1 [18], an integrin-linked kinase. Both the canonical WNT pathway and TGF-β1 require activation of β-catenin, in addition to the E-cadherin/β-catenin complexes localised to epithelial intercellular junctions, implicating both β-catenin and the WNT pathway in the regulation of the EMT [19]. Furthermore, GSK3B, the protein responsible for phosphorylation of the β-catenin molecule and its subsequent proteosomal degradation, has been shown to prevent transition to a mesenchymal phenotype in human embryonic stem cells [20]. Several WNT ligands, FZD receptors and β-catenin have all been reported to be differentially expressed in the unilateral ureteral obstructed (UUO) mouse model of renal injury [4]. In addition, Dickkopf-1 (DKK1), a WNT signalling antagonist, was shown to promote hyperglycaemia-induced mesangial matrix expansion in rat mesangial cells [5].

Previously, common variants within four key genes in the WNT pathway have been investigated for association with diabetic nephropathy [21]. In the present study, a more comprehensive assessment was undertaken of common variants in multiple genes within the WNT pathway. Due to the large number of WNT pathway genes (>65), eight potential candidate genes were chosen on the basis of single nucleotide polymorphisms (SNPs) reaching a nominal significance threshold of 0.05 from the meta-analysed Genetics of Nephropathy–an International Effort (GENIE) Consortium dataset (Table 1) [22]. The chosen SNPs also showed a consistent direction of effect in each of the three case–control collections represented by the GENIE Consortium meta-analysed dataset, an international collaboration of three cohorts of type 1 diabetic patients discordant for DN totalling 2916 with nephropathy and 3315 without nephropathy [22]. Three additional genes, *CTNNB1*, *WNT5A* and *WNT6*, were also included within the analysis despite failing to meet the inclusion criteria, on the basis of previous suggestion of their involvement in the pathogenesis of DN. Although the genotyping platforms used to determine the GENIE data provided reasonable coverage across the potential genes of interest, additional informative haplotype tagging SNPs identified through CEU participant data from HapMap offers a more comprehensive evaluation of any potential genetic effect.

**Table 1:** **Genes included within the analysis on the basis of significant association (P < 0.05) within the GENIE meta-analysis**[22]**and demonstrating a consistent direction of effect across all four cohorts**

**Chromosome**	**Position**	**Gene**	**SNP**	**Direction**	**Allele**	**P-value**
2	219395841	*WNT6*	rs6436094	- + ??	A	0.19
2	219411542	*WNT6*	rs730947	- + −+	A	0.12
2	219427976	*WNT6*	rs7596898	?-??	T	0.73
2	219428221	*WNT6*	rs940469	+ − ++	T	0.11
2	219429434	*WNT6*	rs11695967	+ − ++	T	0.11
2	219434742	*WNT6*	rs10193725	- + −−	T	0.18
2	219440386	*WNT6*	rs6754599	- + −+	C	0.36
2	219452118	*WNT6*	rs3806557	+−−-	A	0.95
2	219454805	*WNT6*	rs10177996	-+++	T	0.95
2	219458990	*WNT6*	rs2385199	-+++	A	0.94
2	219464627	*WNT6*	rs7349332	++−−	T	0.61
3	41205327	*CTNNB1*	rs2691678	-- + −	A	0.32
3	41205658	*CTNNB1*	rs7630377	-- + −	T	0.58
3	41209520	*CTNNB1*	rs9859392	++ − +	C	0.39
3	41215181	*CTNNB1*	rs3864004	----	A	0.38
3	41218746	*CTNNB1*	rs3915129	++++	T	0.38
3	41229650	*CTNNB1*	rs2140090	----	T	0.38
3	41236983	*CTNNB1*	rs1798802	-- + −	A	0.59
3	41237448	*CTNNB1*	rs13072632	++++	T	0.38
3	41243358	*CTNNB1*	rs11564447	+ − +−	T	0.41
3	41247085	*CTNNB1*	rs1880481	----	A	0.37
3	41249076	*CTNNB1*	rs11564450	++++	C	0.37
3	41254444	*CTNNB1*	rs4135385	+−−+	A	0.48
3	41259540	*CTNNB1*	rs9883073	-- + −	A	0.41
3	41262022	*CTNNB1*	rs11711946	++ − +	T	0.41
3	41270771	*CTNNB1*	rs11129896	++++	T	0.20
3	55450313	*WNT5A*	rs815533	-++−	A	0.73
3	55456115	*WNT5A*	rs3913369	- + −−	T	0.10
3	55462468	*WNT5A*	rs751194	-- + −	A	0.15
3	55464838	*WNT5A*	rs1499890	-- + −	A	0.15
3	55466053	*WNT5A*	rs629537	+−−-	A	0.82
3	55466476	*WNT5A*	rs503022	+−−-	A	0.76
3	55467366	*WNT5A*	rs645486	+ − +−	A	0.46
3	55468554	*WNT5A*	rs476986	--++	A	0.47
3	55469119	*WNT5A*	rs6792802	-- + −	T	0.07
3	55471300	*WNT5A*	rs11706227	+ − ++	T	0.07
3	55472993	*WNT5A*	rs11710229	- + −−	A	0.06
3	55474704	*WNT5A*	rs12497254	-- + −	A	0.08
3	55475448	*WNT5A*	rs10865994	-- + −	A	0.07
3	55476215	*WNT5A*	rs1829556	++ − +	T	0.08
3	55482321	*WNT5A*	rs556874	-++−	T	0.13
3	55486715	*WNT5A*	rs472631	+−−-	A	0.73
3	55494897	*WNT5A*	rs648872	+ − ++	A	0.77
3	55495818	*WNT5A*	rs566926	+ − ++	T	0.59
3	55506307	*WNT5A*	rs557077	+−−-	A	0.63
3	55506999	*WNT5A*	rs1160047	---+	A	0.39
3	121176301	*GSK3B*	rs6771023	++++	T	0.05
3	121225411	*GSK3B*	rs6770314	----	A	0.04
3	121232101	*GSK3B*	rs9851174	----	A	0.04
3	121246776	*GSK3B*	rs7652172	----	T	0.04
3	121255730	*GSK3B*	rs968824	++++	A	0.04
3	121273402	*GSK3B*	rs334536	++++	A	0.05
3	121274994	*GSK3B*	rs334535	----	T	0.05
3	121292295	*GSK3B*	rs334559	----	A	0.05
4	108110470	*DKK2*	rs17509845	++++	A	0.01
7	120715028	*WNT16*	rs2952556	----	A	<0.01
7	120723242	*WNT16*	rs10241888	----	A	<0.01
7	120727672	*WNT16*	rs2707521	----	T	0.01
7	120732453	*WNT16*	rs1547960	++++	A	<0.01
7	120739818	*WNT16*	rs10231005	----	A	<0.01
14	58700292	*DAAM1*	rs6573249	----	T	0.04
14	58728544	*DAAM1*	rs1252906	++++	A	0.02
14	58735687	*DAAM1*	rs8016570	++++	T	0.03
14	58744616	*DAAM1*	rs2146009	++++	A	0.01
14	58748939	*DAAM1*	rs1252989	++++	A	0.01
14	58753987	*DAAM1*	rs1270988	++++	A	0.01
14	58795556	*DAAM1*	rs17095967	++++	A	0.02
14	58800998	*DAAM1*	rs1253005	----	T	0.04
14	58847352	*DAAM1*	rs7149497	----	A	0.02
14	58870553	*DAAM1*	rs1957409	----	A	0.03
16	312598	*AXIN1*	rs3916990	++++	A	0.03
16	68144622	*NFAT5*	rs17230557	++++	T	0.02
16	68184592	*NFAT5*	rs17231138	++++	A	0.01
16	68198345	*NFAT5*	rs17297088	----	T	0.02
16	68200854	*NFAT5*	rs17297207	++++	A	0.02
16	68207765	*NFAT5*	rs17231474	++++	A	0.02
16	68224440	*NFAT5*	rs6499238	----	T	0.02
16	68262239	*NFAT5*	rs1437137	----	A	0.02
16	68309874	*NFAT5*	rs689453	----	T	0.02
17	42221887	*WNT3*	rs199496	++++	A	0.01
17	42223260	*WNT3*	rs11655598	++++	C	0.05
17	42223596	*WNT3*	rs199495	----	A	0.02
17	42229159	*WNT3*	rs11650531	++++	T	0.04
19	51811750	*CALM3*	rs11083840	++++	T	0.03

Direction of effect represents that observed in the FinnDiane, UK-ROI, US GoKinD George Washington University, US GoKinD Joslin Diabetes Center.? signifies where a marker failed QC in the original collection and was therefore unavailable for meta-analysis.

The P value presented represents the association of the meta-analysed data from all four GENIE cohorts.

## Methods

### Participants

Research ethics approval was obtained from the South and West Multicentre Research Ethics Committee (MREC/98/6/71) and Queens University Belfast Research Ethics Committee, and written informed consent obtained prior to participation. All recruited individuals were white, had type 1 diabetes mellitus (T1D) diagnosed before 32 years of age and were born in the UK or Ireland. Cases with nephropathy (n = 718) and controls without nephropathy (n = 749) were from the Warren 3/UK Genetics of Kidneys in Diabetes (GoKinD) and all-Ireland collections [23]. The definition of DN in cases was based on development of persistent proteinuria (>0.5g protein/24h) at least 10 years after diagnosis of T1D, hypertension (blood pressure > 135/85 mmHg or treatment with antihypertensive agents) and associated diabetic retinopathy. Controls were individuals with T1D for at least 15 years with normal urinary albumin excretion rates and no evidence of microalbuminuria on repeated testing (at least 3 assays measuring albumin excretion over a minimum 12 month period, with each test separated by at least 3 months). In addition, control subjects had not been prescribed antihypertensive drug treatment avoiding possible misclassification of diabetic individuals with nephropathy as ‘control phenotypes’ when the use of antihypertensive treatment may have reduced urinary albumin excretion into the normal range. Individuals with micro-albuminuria were excluded from both case and control groups since it is not possible to confidently assign a case or control status to such individuals as their urinary albumin excretion may either regress or progress over time [24].

### Haplotype definition, SNP selection and genotyping

A total of 11 genes (*AXIN1, CALM3, CTNNB1, DAAM1, DKK2, GSK3B, NFAT5, WNT3, WNT5A, WNT6* and *WNT16*) were chosen for genotyping (Table 1). SNPs were selected from within these 11 genes to tag common haplotypes (>5% frequency within the HapMap CEU population). Haplotypes for each gene investigated were selected from Phase III, release 2 HapMap (http://www.hapmap.org) CEPH data (Utah residents with ancestry in northern and western Europe; CEU) using Haploview (http://www.broadinstitute.org/haploview) to visualise common haplotypes. Haplotypes were defined using the confidence interval method in Haploview as described in Gabriel *et al*. [25]. Adjacent haplotypes that had a multi-allelic D-prime of greater 0.9 were combined in an iterative fashion. SNPs were selected using multi-marker tagging for their ability to tag unique haplotypes with *r*^*2*^ > 0.8 (LOD threshold 3.0). All SNPs had a minor allele frequency (MAF) ≥5%, with quality control filters of genotype call rate ≥95%, and no deviation from Hardy–Weinberg equilibrium (HWE; *P* < 0.001).

Genotyping was performed by MassARRAY iPLEX (Sequenom, San Diego, CA, USA) or Taqman 5' nuclease (Applied Biosystems, Foster City, CA, USA) assays according to the manufacturers’ instructions. DNA samples were excluded if missing genotypes exceeded 10%. Other quality control measures included parent/offspring trio samples, duplicates on plates, random sample allocation to plates, independent scoring of problematic genotypes by two individuals and re-sequencing of selected DNAs to validate genotypes.

### Statistical analysis

Clinical characteristics of cases and controls were compared using the z-test for large independent samples and the χ^2^ test. Association analyses were performed using PLINK [26]. Initially a χ^2^ test for trend (1 *df*) was used with adjustment for collection centre. Logistic regression analysis was then performed on each SNP with terms for potential confounders (collection centre, gender, duration of T1D and HbA1c) included in the model. The level of statistical significance was set at 5% with correction for multiple testing performed by permutation test (n = 100,000). Pairwise interactions between SNPs were tested in the statistical programming package R, using logistic regression to compare models with and without the interaction terms to obtain a likelihood ratio test. The results of the interaction analysis were corrected for multiple testing by false discovery rate (FDR < 5%).

## Results and discussion

A total of 90 SNPs were genotyped, 85 using MassARRAY iPLEX Gold technology (Sequenom, San Diego, CA, USA), and five using Taqman 5’ nuclease assay (Applied Biosystems, Foster City, CA, USA) in 719 cases and 748 controls. Quality criteria were applied to the data before association analysis. A total of 35 individuals with more than 10% missing genotype data were removed from the analysis. All SNPs passed the genotyping and Hardy-Weinberg thresholds of 95% and *P* < 0.001 respectively. No Mendelian errors or inconsistencies between duplicate samples were observed. The final average genotyping rate was 98.9% in 700 cases, and 732 controls.

The clinical characteristics of the DN cases (n = 700) and diabetic controls (n = 732) genotyped in this study, which met quality control filters, are listed in Table 2. There were more males, higher mean HbA1c and blood pressure values (despite the use of antihypertensive treatment) in the case group compared with the control group. All comparisons were significant at *P* < 0.001 with the exception of age at diagnosis which did not differ significantly between groups. Approximately one quarter of cases (26.6%) had ESRD.

**Table 2:** Clinical characteristics of the genotyped diabetic nephropathy (DN) cases and diabetic controls

**Characteristic**	**DN Case (n = 700)**	**Control (n = 732)**
Male; n (%)	405 (57.8%)	306 (41.8%)
Age at diagnosis of T1D (yr)	14.7 ± 7.6	15.4 ± 7.9
Duration of T1D (yr)^a^	33 ± 9.3	28.1 ± 9
HbA1c (%)^b^	9 ± 1.9	8.6 ± 1.5
Systolic blood pressure (mmHg)^b^	144.9 ± 20.9	125.1 ± 14.8
Diastolic blood pressure (mmHg)^b^	81.5 ± 11.4	75.5 ± 7.7
Body mass index (kg/m^2^)	26.3 ± 4.8	26.1 ± 4.2
Serum cholesterol (mmol/L)	5.3 ± 1.2	5.1 ± 0.9
Serum creatinine (umol/L)^c^; median (interquartile range)	130 (102–182.5)	92 (78.8 - 105)
Glomerular filtration rate (ml/min/1.73m^2^)^c^; median (interquartile range)	47.8 (33.6 - 66.2)	70 (59.3 - 85.5)
End-stage renal disease n (%)	186 (26.6%)	NA

Unless otherwise stated values are mean ± standard deviation.

^a^Calculated from the dates of diagnosis and recruitment.

^b^Average of the three most recent values prior to recruitment.

^c^Excludes subjects receiving renal replacement therapy (dialysis or transplant).

*P* < 0.05 for age at diagnosis; *P* <0.001 for all other comparisons except body mass index.

T1D = type 1 diabetes.

SNPs chosen to tag common haplotypes across the 11 genes selected on the basis of their significant and common direction of effect across the GENIE cohorts (Table 1) [22] were assessed by logistic regression analysis with adjustment for collection centre, gender, duration of T1D and HbA1c (Table 3). Twenty-six putative linkage disequilibrium (LD) blocks were identified across the 11 genes, yielding 110 common haplotypes with an estimated frequency >5%. None of the haplotypes examined were significantly associated with DN at *P* < 0.01, however eight haplotypes were significantly associated with DN at *P* < 0.05. Of the eight haplotypes, three were in *GSK3B*, two in *AXIN1*, two in *DAAM1*, and one in *NFAT5*. However, no significant association between haplotype and DN remained after correction for multiple testing (data not shown).

**Table 3:** Minor allele frequencies, genotype counts and logistic regression model adjusted for collection centre, gender, duration of type 1 diabetes, and average HbA1c

**Chr**^**a**^	**Position**	**Gene**	**SNP ID**	**Allele**	**Case count**	**MAF**	**Control count**	**MAF**	**OR**^**b**^	**C.I.**^**c**^	**P**^**d**^
2	219428221	*WNT6*	rs940469	[T/C]	24/170/483	0.16	24/170/483	0.18	0.86	0.67 - 1.11	0.25
2	219430908	*WNT6*	rs690877	[T/C]	56/276/360	0.28	56/276/360	0.27	0.92	0.74 - 1.14	0.43
2	219440386	*WNT6*	rs6754599	[G/C]	18/166/494	0.15	18/166/494	0.17	0.85	0.65 - 1.11	0.22
2	219458990	*WNT6*	rs2385199	[G/A]	23/200/471	0.18	23/200/471	0.19	0.90	0.7 - 1.16	0.43
3	41205327	*CTNNB1*	rs2691678	[C/T]	51/250/390	0.25	51/250/390	0.25	1.01	0.81 - 1.26	0.91
3	41243358	*CTNNB1*	rs11564447	[G/T]	1/59/634	0.04	1/59/634	0.04	0.91	0.57 - 1.43	0.68
3	41254444	*CTNNB1*	rs4135385	[G/A]	49/237/398	0.24	49/237/398	0.23	1.04	0.84 - 1.3	0.70
3	55450313	*WNT5A*	rs815533	[A/G]	22/206/466	0.18	22/206/466	0.17	1.27	0.99 - 1.64	0.06
3	55456115	*WNT5A*	rs3913369	[T/G]	33/261/394	0.24	33/261/394	0.23	0.91	0.72 - 1.14	0.40
3	55466053	*WNT5A*	rs629537	[A/G]	11/145/534	0.12	11/145/534	0.13	0.89	0.67 - 1.18	0.40
3	55467090	*WNT5A*	rs845542	[C/T]	44/272/377	0.26	44/272/377	0.25	1.19	0.95 - 1.49	0.13
3	55494897	*WNT5A*	rs648872	[T/C]	13/172/501	0.14	13/172/501	0.17	0.86	0.67 - 1.1	0.22
3	55495818	*WNT5A*	rs566926	[T/G]	49/252/368	0.26	49/252/368	0.27	0.99	0.8 - 1.24	0.96
3	121016036	*GSK3B*	rs11917714	[T/C]	19/205/470	0.18	19/205/470	0.16	1.02	0.79 - 1.31	0.89
3	121018485	*GSK3B*	rs11929668	[G/C]	22/170/501	0.15	22/170/501	0.18	0.87	0.67 - 1.12	0.27
3	121133839	*GSK3B*	rs17204365	[C/T]	2/41/648	0.03	2/41/648	0.03	1.61	0.91 - 2.85	0.10
3	121157741	*GSK3B*	rs17810235	[T/C]	74/303/312	0.33	74/303/312	0.29	1.33	1.09 - 1.64	0.01
3	121300363	*GSK3B*	rs17471	[A/T]	3/110/581	0.08	3/110/581	0.09	0.67	0.47 - 0.95	0.02
3	121306727	*GSK3B*	rs11927974	[A/G]	8/101/584	0.08	8/101/584	0.09	0.98	0.71 - 1.35	0.89
3	121307901	*GSK3B*	rs334538	[A/G]	22/191/481	0.17	22/191/481	0.19	0.89	0.7 - 1.14	0.35
3	121308854	*GSK3B*	rs12053912	[T/A]	17/186/485	0.16	17/186/485	0.14	1.21	0.92 - 1.58	0.18
3	121315311	*GSK3B*	rs334543	[C/A]	66/311/307	0.32	66/311/307	0.35	0.79	0.65 - 0.97	0.02
4	108055760	*DKK2*	rs429941	[G/A]	53/282/356	0.28	53/282/356	0.28	1.08	0.88 - 1.34	0.46
4	108062942	*DKK2*	rs10488898	[A/G]	2/77/603	0.06	2/77/603	0.07	0.85	0.58 - 1.24	0.40
4	108065243	*DKK2*	rs17037102	[A/G]	7/132/526	0.11	7/132/526	0.10	1.17	0.85 - 1.6	0.34
4	108084260	*DKK2*	rs10488899	[G/T]	42/237/410	0.23	42/237/410	0.21	1.10	0.88 - 1.39	0.40
4	108110470	*DKK2*	rs17509845	[A/G]	1/66/622	0.05	1/66/622	0.05	1.08	0.69 - 1.7	0.74
4	108120464	*DKK2*	rs17618172	[G/A]	20/219/445	0.19	20/219/445	0.20	1.01	0.79 - 1.28	0.96
4	108135958	*DKK2*	rs7690634	[A/T]	9/165/515	0.13	9/165/515	0.12	1.19	0.89 - 1.58	0.24
4	108165736	*DKK2*	rs4956277	[C/A]	30/246/396	0.23	30/246/396	0.24	0.94	0.75 - 1.17	0.58
4	108170809	*DKK2*	rs10021344	[G/A]	150/357/185	0.47	150/357/185	0.48	0.99	0.82 - 1.19	0.88
4	108173328	*DKK2*	rs956137	[A/C]	4/111/577	0.09	4/111/577	0.08	1.28	0.92 - 1.8	0.15
4	108173463	*DKK2*	rs6827902	[C/A]	28/237/416	0.22	28/237/416	0.21	1.15	0.91 - 1.45	0.23
4	108181414	*DKK2*	rs6823507	[C/T]	1/88/593	0.07	1/88/593	0.08	0.87	0.6 - 1.26	0.46
4	108185493	*DKK2*	rs419178	[C/T]	38/272/379	0.25	38/272/379	0.27	0.86	0.69 - 1.07	0.17
4	108199999	*DKK2*	rs398093	[T/C]	12/138/510	0.12	12/138/510	0.12	1.09	0.81 - 1.46	0.58
4	108200154	*DKK2*	rs17510191	[C/T]	20/251/423	0.21	20/251/423	0.18	1.13	0.89 - 1.43	0.31
7	120727672	*WNT16*	rs2707521	[T/C]	103/338/239	0.40	103/338/239	0.41	0.90	0.74 - 1.1	0.32
7	120738146	*WNT16*	rs13247600	[C/G]	3/81/610	0.06	3/81/610	0.07	1.08	0.74 - 1.57	0.69
7	120738380	*WNT16*	rs2707520	[G/T]	166/340/183	0.49	166/340/183	0.49	1.12	0.92 - 1.35	0.25
7	120741508	*WNT16*	rs983926	[A/G]	6/101/587	0.08	6/101/587	0.09	0.93	0.67 - 1.3	0.68
7	120751201	*WNT16*	rs3757552	[C/T]	9/151/534	0.12	9/151/534	0.12	0.89	0.67 - 1.19	0.44
7	120762001	*WNT16*	rs3801387	[C/T]	69/283/340	0.30	69/283/340	0.29	1.00	0.81 - 1.23	0.99
7	120774586	*WNT16*	rs2707461	[T/C]	18/187/489	0.16	18/187/489	0.17	0.98	0.77 - 1.26	0.89
14	58696138	*DAAM1*	rs8016068	[T/C]	4/176/514	0.13	4/176/514	0.12	1.09	0.81 - 1.47	0.57
14	58713714	*DAAM1*	rs17095819	[G/A]	15/185/477	0.16	15/185/477	0.13	1.28	0.96 - 1.71	0.09
14	58728544	*DAAM1*	rs1252906	[G/T]	19/164/511	0.15	19/164/511	0.18	0.75	0.58 - 0.97	0.03
14	58826755	*DAAM1*	rs7155987	[T/C]	18/180/496	0.16	18/180/496	0.14	1.10	0.84 - 1.44	0.48
14	58833620	*DAAM1*	rs12878138	[T/C]	2/99/593	0.07	2/99/593	0.07	0.96	0.67 - 1.39	0.84
14	58861382	*DAAM1*	rs941882	[C/A]	40/238/416	0.23	40/238/416	0.23	1.03	0.82 - 1.28	0.82
14	58885779	*DAAM1*	rs17834014	[G/A]	3/86/605	0.07	3/86/605	0.08	0.97	0.67 - 1.42	0.88
14	58886484	*DAAM1*	rs12880248	[A/G]	12/177/505	0.14	12/177/505	0.14	1.06	0.8 - 1.39	0.70
14	58889427	*DAAM1*	rs12878070	[G/T]	150/334/210	0.46	150/334/210	0.42	1.10	0.91 - 1.32	0.33
14	58908520	*DAAM1*	rs17096179	[C/A]	5/104/585	0.08	5/104/585	0.09	0.88	0.63 - 1.24	0.47
14	58913159	*DAAM1*	rs1253192	[A/G]	5/80/609	0.06	5/80/609	0.09	0.65	0.46 - 0.93	0.02
16	275374	*AXIN1*	rs2685127	[T/C]	7/157/529	0.12	7/157/529	0.13	1.02	0.76 - 1.36	0.92
16	276397	*AXIN1*	rs400037	[T/C]	32/243/410	0.22	32/243/410	0.22	0.97	0.77 - 1.21	0.77
16	281080	*AXIN1*	rs12925669	[A/G]	22/171/501	0.15	22/171/501	0.16	1.06	0.82 - 1.37	0.65
16	289294	*AXIN1*	rs214246	[C/T]	148/349/196	0.47	148/349/196	0.49	0.93	0.77 - 1.12	0.45
16	303385	*AXIN1*	rs12930863	[C/T]	20/175/490	0.16	20/175/490	0.16	1.05	0.81 - 1.36	0.70
16	313818	*AXIN1*	rs11646942	[A/C]	51/261/355	0.27	51/261/355	0.31	0.89	0.72 - 1.1	0.29
16	333343	*AXIN1*	rs395901	[A/G]	7/115/569	0.09	7/115/569	0.09	0.82	0.6 - 1.14	0.24
16	336265	*AXIN1*	rs1805105	[A/G]	67/310/284	0.34	67/310/284	0.31	1.19	0.97 - 1.47	0.10
16	68112382	*NFAT5*	rs12921716	[A/G]	6/113/575	0.09	6/113/575	0.08	0.95	0.68 - 1.32	0.75
16	68117197	*NFAT5*	rs4783720	[C/T]	22/184/488	0.16	22/184/488	0.15	1.10	0.85 - 1.43	0.47
16	68200854	*NFAT5*	rs17297207	[G/A]	3/69/612	0.05	3/69/612	0.07	0.66	0.44 - 0.98	0.04
16	68203623	*NFAT5*	rs11639947	[T/C]	22/183/467	0.17	22/183/467	0.21	0.80	0.63 - 1.02	0.07
16	68281721	*NFAT5*	rs1064825	[A/G]	0/74/619	0.05	0/74/619	0.06	0.84	0.55 - 1.28	0.42
16	68290961	*NFAT5*	rs7359336	[G/A]	128/329/227	0.43	128/329/227	0.41	1.04	0.86 - 1.26	0.69
17	42163544	*WNT3*	rs35937770	[A/G]	83/321/283	0.35	83/321/283	0.35	0.99	0.81 - 1.21	0.90
17	42188384	*WNT3*	rs35732828	[A/C]	19/200/466	0.17	19/200/466	0.17	1.05	0.81 - 1.35	0.71
17	42209035	*WNT3*	rs199520	[G/A]	38/237/419	0.23	38/237/419	0.22	1.04	0.83 - 1.3	0.75
17	42221887	*WNT3*	rs199496	[A/G]	5/117/569	0.09	5/117/569	0.07	1.11	0.78 - 1.58	0.56
17	42221965	*WNT3*	rs11658976	[G/A]	129/338/227	0.43	129/338/227	0.42	1.03	0.85 - 1.25	0.74
17	42223260	*WNT3*	rs11655598	[G/C]	46/249/387	0.25	46/249/387	0.29	0.94	0.76 - 1.16	0.57
17	42223353	*WNT3*	rs12452064	[A/G]	147/322/211	0.45	147/322/211	0.43	1.08	0.9 - 1.3	0.42
17	42224733	*WNT3*	rs10432043	[T/C]	86/301/284	0.35	86/301/284	0.33	1.07	0.88 - 1.31	0.50
17	42234514	*WNT3*	rs7207916	[A/G]	113/313/267	0.39	113/313/267	0.43	0.93	0.77 - 1.12	0.43
17	42243374	*WNT3*	rs11079737	[A/G]	54/268/338	0.28	54/268/338	0.27	1.12	0.91 - 1.38	0.30
17	42259048	*WNT3*	rs11079738	[G/A]	143/362/188	0.47	143/362/188	0.47	1.02	0.84 - 1.23	0.88
17	42261948	*WNT3*	rs8069437	[T/C]	35/225/433	0.21	35/225/433	0.23	0.85	0.68 - 1.07	0.16
19	51775092	*CALM3*	rs8103534	[C/T]	63/298/333	0.31	63/298/333	0.31	0.96	0.78 - 1.18	0.67
19	51781925	*CALM3*	rs4274528	[T/C]	7/144/542	0.11	7/144/542	0.12	1.17	0.87 - 1.57	0.31
19	51790267	*CALM3*	rs7260181	[C/T]	146/355/193	0.47	146/355/193	0.46	1.08	0.89 - 1.31	0.43
19	51798474	*CALM3*	rs4380146	[G/T]	69/284/327	0.31	69/284/327	0.32	1.03	0.84 - 1.26	0.76
19	51800325	*CALM3*	rs11671131	[C/T]	12/167/513	0.14	12/167/513	0.14	0.87	0.67 - 1.14	0.32
19	51804580	*CALM3*	rs710889	[A/G]	94/315/285	0.36	94/315/285	0.36	0.93	0.76 - 1.13	0.47
19	51804978	*CALM3*	rs10405893	[A/G]	5/153/535	0.12	5/153/535	0.11	1.25	0.92 - 1.69	0.16
19	51811750	*CALM3*	rs11083840	[G/T]	118/341/235	0.42	118/341/235	0.44	0.91	0.75 - 1.1	0.32
19	51811873	*CALM3*	rs11083841	[G/A]	26/181/457	0.18	26/181/457	0.16	1.15	0.9 - 1.47	0.26

^a^Chr = Chromosome.

^b^OR = Odds Ratio.

^c^C.I. = 95% Confidence Interval.

^d^P-value, not corrected for multiple testing.

In a single marker analysis, adjusted by collection centre, no SNPs were associated with DN at *P* < 0.01 (the significance threshold set was corrected for multiple testing), however five SNPs, rs17810235, rs11639947, rs11646942, rs17095819, and rs17510191 in *GSK3B*, *NFAT5*, *AXIN1*, *DAAM1*, *DKK2* had *P*-values <0.05 as shown in Table 4a. Logistic regression analyses were performed with adjustment for collection centre, gender, duration of T1D, and average HbA1c as covariates in the model. The most significant association was reported for rs17810235 in *GSK3B* (Table 4b; P = 0.006). Five additional SNPs demonstrated a *P* <0.05, although they were not supported in the univariate analysis alone. Although limited in power, a subgroup analysis defined by comparison of ESRD as the primary phenotype versus non-ESRD, identified two significantly associated SNPs, rs1253192 and rs11079737 in *DAAM1* and *WNT3* respectively with P = 0.009, although neither association survived correction for multiple testing (Table 4c).

**Table 4:** Most significant results from logistic regression analyses

**a. Single SNP analysis for diabetic nephropathy**				
**Gene**	**SNP**	^**a**^**OR**	^**b**^**C.I.**	^**c**^**P**
*GSK3B*	rs17810235	1.23	1.03 - 1.45	0.022
*NFAT5*	rs11639947	0.79	0.64 - 0.97	0.023
*AXIN1*	rs11646942	0.82	0.68 - 0.97	0.026
*DAAM1*	rs17095819	1.27	1.01 - 1.59	0.045
*DKK2*	rs17510191	1.23	1.01 - 1.49	0.047
**b. Adjusted logistic regression analysis for diabetic nephropathy**				
*GSK3B*	rs17810235	1.33	1.09 - 1.64	0.006
*DAAM1*	rs1253192	0.65	0.46 - 0.93	0.018
*GSK3B*	rs17471	0.67	0.47 - 0.95	0.025
*GSK3B*	rs334543	0.79	0.65 - 0.97	0.025
*DAAM1*	rs1252906	0.75	0.58 - 0.97	0.027
*NFAT5*	rs17297207	0.66	0.44 - 0.98	0.040
**c. Adjusted logistic regression analysis for end-stage renal disease**				
*WNT3*	rs11079737	1.50	1.13 - 1.98	0.005
*DAAM1*	rs1253192	0.43	0.23 - 0.81	0.009
*DAAM1*	rs941882	1.45	1.08 - 1.94	0.013
*CALM3*	rs8103534	0.68	0.50 - 0.92	0.013
*DKK2*	rs6827902	1.47	1.08 - 1.99	0.015
*DAAM1*	rs1252906	0.60	0.40 - 0.91	0.015
*WNT3*	rs11079738	1.33	1.02 - 1.74	0.035
*DAAM1*	rs7155987	1.43	1.02 - 2.02	0.040
*DAAM1*	rs17834014	1.61	1.01 - 2.55	0.045
*WNT6*	rs940469	0.68	0.47 - 0.99	0.046

^a^OR: Odds Ratio.

^b^C.I.: 95% Confidence Intervals.

^c^P: value adjusted for collection centre, gender, duration of type 1 diabetes, and average HbA1c as covariates; not corrected for multiple testing.

Assessment of 4005 pair-wise interactions between the 90 SNPs was performed by logistic regression analyses with adjustment for collection centre, gender, duration of T1D, and HbA1c. The χ^2^ likelihood ratio tests (Table 5) identified four interaction terms as nominally significant between SNPs *in AXIN1, DAAM1, DKK2, WNT3* and *WNT6*. However, these interactions were not significant following correction for multiple testing by FDR *P* < 5%.

**Table 5:** Interaction analyses with adjustment for covariates

**Gene1**	**SNP1**	**Gene2**	**SNP2**	**P**	^**a**^**P**
*AXIN1*	rs395901	AXIN1	rs1805105	8.40E-05	0.39
*WNT6*	rs690877	WNT3	rs199496	0.00043	0.58
*DAAM1*	rs17834014	WNT3	rs199496	0.00049	0.70
*DKK2*	rs398093	DAAM1	rs17834014	0.00072	0.73
*GSK3B*	rs11917714	DKK2	rs17510191	0.00096	0.73

^a^Corrected for multiple testing by False Discovery Rate.

There is an increasing body of evidence to suggest that modulation of the WNT pathways may play a role in the development of DN. β-catenin and TCF/LEF have been shown to directly induce the expression of the cyclic nucleotide phosphodiesterase CNP, a regulator of fibroblast proliferation and activation [2]. The expression of secreted frizzled-related protein 4 (*SFRP4*), an inhibitor of WNT signalling, is decreased following UUO. This decrease is concomitant with increased levels of WNT/β-catenin signalling, in tubular and interstitial cells, along with increased fibronectin and smooth muscle actin, both markers of fibrosis. Introduction of recombinant *SFRP4* reduced the markers of fibrosis and WNT/β-catenin signalling. Furthermore E-cadherin expression was partially maintained by treatment with recombinant *SFRP4*, and the number of myofibroblasts decreased [3]. DKK1 is shown to be increased in mesangial cells in response to stimulation with high concentrations of glucose [5]. In addition high concentrations of glucose decreased WNT signalling and increased TGF-β1 and fibronectin expression in mesangial cells. Transfection of WNT4, WNT5a, GSK3β and β-catenin ameliorated the TGF-β1-induced fibrosis [8]. Cultured podocytes with stabilised β-catenin are less motile and less adherent to the extracellular matrix whereas deletion of β-catenin rendered the cells more susceptible to apoptosis [6].

Gene-based assessments of association are increasingly been viewed as a useful complement to genome-wide association studies (GWAS) [27]. The gene-based approach reduces the problems associated with multiple testing that inhibit GWAS by reducing the number of statistical tests under consideration. Our study has adopted a two stage approach to evaluate common variants in all WNT pathway members in relation to DN. SNPs located in genes implicated in the WNT pathways that failed to demonstrate significant association and direction of effect across all GENIE cohorts [22] were excluded at the first step. WNT pathway members that demonstrated significant association and direction of effect with DN across the three GENIE case–control collections were then evaluated more meticulously through refined genotyping of haplotype tagging SNPs. This approach offers a more comprehensive assessment of common variants across the WNT pathways in comparison to previously published studies. Univariate SNP analysis failed to identify any association with DN. Multivariate regression analyses of common haplotypic structure also failed to reveal any associations that remained significant after correction for multiple testing. All possible combinations of pair-wise SNP-SNP interactions were tested as an interaction term in a logistic regression model. Due to the large number of tests, and the unsuitability of permutations as a correction for multiple testing in interaction analyses, the false discovery rate method was used, although no associations remained significant after correction.

There are a number of inherent limitations associated with using a restricted number of SNPs across a chosen set of genes [28]: (1) identification of association does not necessarily equate to functional significance given the concept of LD. (2) assessing one or two SNPs per gene may provide inadequate representation of the genetic architecture at that locus. (3) patterns of LD can vary significantly within and between different populations and therefore a significant association in one population may not necessarily translate across all populations. In addition, common genetic loci are likely to explain only a proportion of the variation contributing to the phenotype under consideration. Evidence in support of rare variants with potentially large individual effect size is currently under investigation in DN. Since our study focused only on common variants, untyped, highly penetrant rare variants in these genes could also contribute to DN. The size of our study was such that it had 90% power to detect variants with odds ratios of 1.69, 1.50, 1.44, and 1.42 and MAF of 10%, 20%, 30%, and 40%, respectively. Sample sizes of the magnitude required to detect variants present in the population with a lower frequency or with a smaller effect size were not available for analysis and would require international, multicentre collaborative efforts. Future amalgamation of independent cohorts with similar DN phenotypes will enable a more robust evaluation of such loci. In addition, other factors such as copy number variation or indeed epigenetic mechanisms (e.g. DNA methylation, histone modification and microRNAs) may also modify gene function and/or expression profiles affecting these pathways and modulating disease risk accordingly.

## Conclusion

There was no association observed between DN and either common variants or haplotypes among any of the genes associated with the WNT pathway. It is unlikely that common variants located within WNT pathway genes have a major role in the underlying genetics of DN. Further investigation of rare variants, copy number variants or epigenetic mechanisms, in WNT pathway members may identify potential risk factors that contribute to the genetic susceptibility of DN, in addition to identifying potential therapeutic targets for this disease.

## Competing interests

The authors declare that they have no competing interests.

## Authors’ contributions

Conceived and designed the experiments: DHK DAS JKC APM GJM. Performed the experiments: DHK. Analyzed the data: DHK CCP GJM. Contributed reagents/materials/analysis tools: AJM DAS APM JKC. Wrote the paper: DHK DAS APM CCP GJM. All authors have approved the final version of the manuscript.

## Pre-publication history

The pre-publication history for this paper can be accessed here:

http://www.biomedcentral.com/1471-2369/14/126/prepub
